# The impact of Hayward green kiwifruit on dietary protein digestion and protein metabolism

**DOI:** 10.1007/s00394-020-02363-5

**Published:** 2020-09-24

**Authors:** Sanghee Park, David D. Church, Carlene Starck, Scott E. Schutzler, Gohar Azhar, Il-Young Kim, Arny A. Ferrando, Paul J. Moughan, Robert R. Wolfe

**Affiliations:** 1 University of Arkansas for Medical Sciences, 4301 West Markham Street, Slot 806, Little Rock, AR 72205-7199 USA; 2 Riddet Institute, Massey University, Palmerston North, New Zealand; 3 Department of Molecular Medicine, Lee Gil Ya Cancer and Diabetes Institute, College of Medicine, Gachon University, Incheon, Republic of Korea

**Keywords:** Essential amino acids, Stable isotope tracers, Muscle protein synthetic rate, Whole-body protein turnover

## Abstract

**Purpose:**

The purpose of the study was to determine if an actinidin protease aids gastric digestion and the protein anabolic response to dietary protein.

**Methods:**

Hayward green kiwifruit (containing an actinidin protease) and Hort 16A gold kiwifruit (devoid of actinidin protease) were given in conjunction with a beef meal to healthy older subjects. Twelve healthy older males (*N* = 6) and females (*N* = 6) were studied with a randomized, double-blinded, crossover design to assess muscle and whole-body protein metabolism before and after ingestion of kiwifruit and 100 g of ground beef. Subjects consumed 2 of each variety of kiwifruit daily for 14 d prior to each metabolic study, and again during each study with beef intake.

**Results:**

Hayward green kiwifruit consumption with beef resulted in a more rapid increase in peripheral plasma essential amino acid concentrations. There were significant time by kiwifruit intake interactions for plasma concentrations of EAAs, branched chain amino acids (BCAAs), and leucine (*P* < 0.01). However, there was no difference in the total amount of EAAs absorbed. As a result, there were no differences between kiwifruit in any of the measured parameters of protein kinetics.

**Conclusion:**

Consumption of Hayward green kiwifruit, with a beef meal facilitates protein digestion and absorption of the constituent amino acids as compared to Hort 16A gold kiwifruit.

**Clinical trial:**

NCT04356573, April 21, 2020 “retrospectively registered”.

## Introduction

Consumption of protein-rich animal food sources, such as beef, pork, and chicken has been recommended by some as a pragmatic approach for older individuals to achieve optimal essential amino acid intake [1]. Whereas the total amount of amino acids absorbed as a result of digestion of animal proteins does not appear to be significantly affected by age [2], a slower digestion of meat in older individuals leads to a feeling of fullness, which in turn may be a factor in the reduction in meat consumption with aging [3]. Moreover, rapidly digestible protein sources have been shown to provide a more positive anabolic response (i.e., net gain of muscle protein) than slowly absorbed protein sources [4]. The importance of speed of digestion is further supported by the observation that minced beef was found to be more anabolic than beef steak [5].

Actinidin, a cysteine protease highly expressed in Hayward green kiwifruit but not in Hort 16A gold kiwifruit, has received attention as a possible aid to the digestion of meats and other food proteins. Actinidin is known to increase the gastric digestion of meat protein, leading to a more rapid gastric emptying of beef proteins in animal models [6, 7]. The improved gastric digestion of meat proteins in monogastric animals suggests that consumption of Hayward green kiwifruit may aid the digestion of meat in humans as well. Improved gastric digestion may have an anabolic effect in older adults by accelerating the appearance of EAAs in the blood.

We hypothesized that consumption of Hayward green kiwifruit (containing actinidin) would speed the digestion of dietary beef consumed at the same time, resulting in a more rapid increase in the rate of appearance of essential amino acids (EAAs) in the blood, as compared to consumption of Hort 16A gold Kiwifruit with beef. We further hypothesized that a more rapid appearance of EAAs in the circulation following the consumption of Hayward green kiwifruit would result in a greater anabolic response to dietary beef.

## Materials and methods

### Subjects

Twelve healthy male and female older adults, between 60 and 85 years of age, were recruited from the Little Rock area utilizing local newspaper advertisements and flyers. Subject eligibility for the study was determined by a battery of tests, including medical history, physical exam and blood panel. Exclusion criteria included unwillingness to eat meat, regular consumption of dietary protein or amino acid supplements, diabetes, active malignancy within the past 6 months, gastrointestinal bypass surgery, a chronic inflammatory disease, use of corticosteroids, and any unstable medical conditions. In addition, subjects who performed any type of regular exercise more than once a week were excluded. All subjects gave their informed consent for inclusion before they participated in the study. The study was conducted in accordance with the Declaration of Helsinki, and the protocol was reviewed and approved by the Institutional Review Board of the University of Arkansas for Medical Sciences, Protocol Number: 206814. Subject characteristics are shown in Table 1.

**Table 1:** Subject characteristics

Characteristics	Hayward green or Hort 16A gold kiwifruit
Male, female	12 (6/6)
Age, years	72.5 ± 1.9
BMI, kg/m^2^	28.7 ± 0.8
LBM, kg	48.5 ± 2.5

### Experimental design

During the screening visit, dual-energy X-ray absorptiometry was performed to determine body composition to express whole body protein kinetics relative to lean body mass. Subjects participated in two feeding studies in a crossover design with a 2 week washout period between experimental periods. In one experimental period, subjects consumed 2 Hayward green kiwifruit per day for 14 days prior to the metabolic study. In the other protocol, subjects consumed 2 Hort 16A gold kiwifruit per day for 14 days prior to the metabolic study. The two-week interval preceding the metabolic study was to adapt subjects to consuming kiwifruit, since kiwifruit are not a normal part of the American diet. Subjects were randomly assigned to consume Hayward green Kiwifruit or Hort 16A gold kiwifruit first. Subjects were asked to complete a diary to document the date and time of their kiwifruit ingestion. The diary was returned to study staff and reviewed to ensure compliance before participation in the tracer study. Diets were not controlled during the two-week intervals in which kiwi fruit were consumed so that any findings would not be limited to a specific diet. Subjects were instructed to abstain from strenuous physical activity for at least 72 h before the metabolic study, since prior exercise amplifies the anabolic response to dietary protein intake [8].

**Fig. 1:**
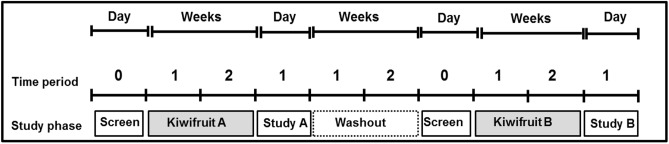
Outline of crossover design showing phases of study for a subject undergoing treatment A first, and treatment B second

A schematic diagram of the experimental design is shown in Fig. 1.

### Stable isotope tracer infusion protocol

**Fig. 2:**
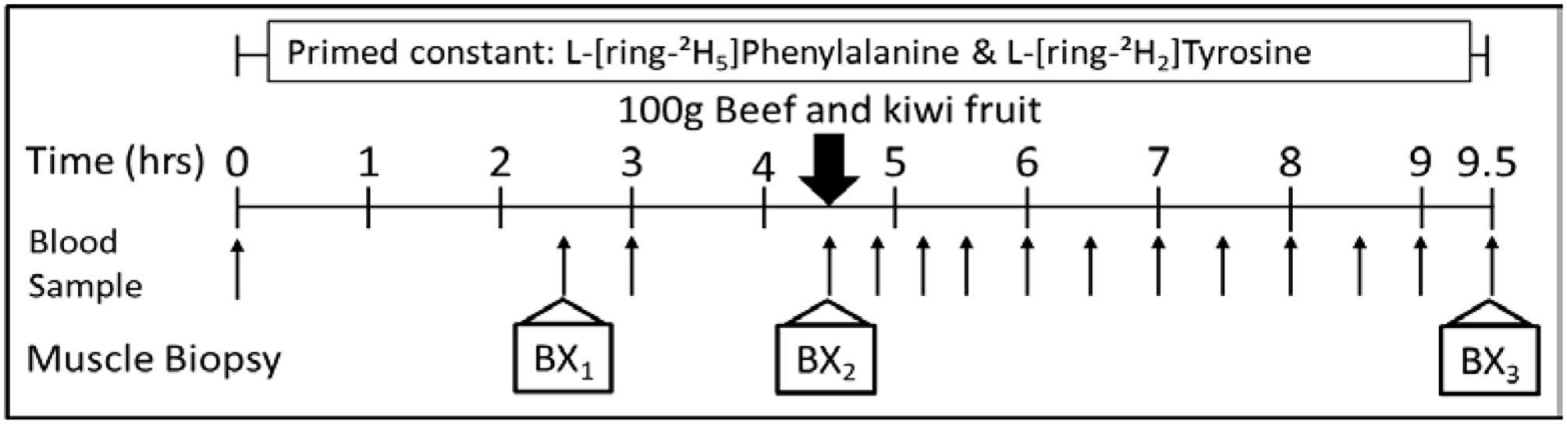
Stable isotope tracer infusion protocol. *BX* biopsy

After vital signs were measured, an IV catheter was inserted into a vein on each arm of the subjects. One catheter was used to infuse the stable isotopes L-ring-^2^H_5_ phenylalanine and ^2^H_2_-tyrosine. The other catheter was used for frequent sampling of arterialized blood after warming the arm by means of a heated box. After an initial blood sample was obtained, the study nurse administered priming doses of the two above isotopes as well as a priming dose of ^2^H_4_-tyrosine. After administration of priming doses, isotopes were infused at constant rates throughout the entire 9.5 h infusion protocol. The infusion rates were: L-ring-^2^H_5_ phenylalanine: 4.6 μmol/kg prime, 3.92 μmol/kg/h infusion; ^2^H_2_-tyrosine: 0.95 μmol/kg prime, 1.57 μmol/kg/h infusion; ^2^H_4_-tyrosine, 0.33 μmol/kg. Each tracer study consisted of a 4.5-h basal fasted period and a 5-h interval following meal ingestion. Blood samples were obtained throughout to measure plasma amino acid concentrations and phenylalanine (Phe) appearance in the blood (Ra Phe). Muscle biopsies were obtained from the vastus lateralis muscle using a Bergstrom needle at 2.5 and 4.5 h after the start of the tracer study to determine the basal rate of muscle protein synthesis. The test meal of two of their group-specific kiwifruit and 100 g of cooked ground beef (85% lean) was served directly after the second muscle biopsy. A third muscle biopsy was obtained at the end of the tracer infusion. Figure 2 shows a schematic representation of the tracer protocol, including the exact times of collection of blood samples and muscle biopsies. At the conclusion of the 9.5-h infusion study, the IV catheters were removed, and the sites dressed with sterile bandages. Written and verbal instructions regarding the care of the muscle biopsy site were provided. A snack and beverage were offered to the subjects, and after vital signs were measured, subjects were free to leave.

### Calculation of protein kinetics

Whole body protein kinetics were calculated based on determinations of the rates of appearance (Ra) of phenylalanine (Phe) and tyrosine (Tyr) into plasma, the fraction of Ra Phe hydroxylated irreversibly to tyrosine, the amount of Phe ingested in the beef meal, and the true ileal digestibility of the amino acids in beef (90%, [9]). Since Phe cannot be produced in the body, Ra Phe in the post-absorptive state is a direct reflection of the rate of protein breakdown. In the steady state, the rate of uptake of Phe (Rd Phe) matches Ra Phe. Rd Phe has only two possible fates: incorporation into protein (protein synthesis) and irreversible hydroxylation to tyrosine. Measurement of Ra Phe (and thus Rd Phe) and the rate of hydroxylation of Phe therefore enables the calculation of the rates of protein breakdown, synthesis and net balance (NB) in the post-absorptive state. In the fed state (following meal ingestion at 4.5 h), Ra Phe is derived from both protein breakdown and absorption of dietary Phe (exogenous Ra Phe). Account must be taken of the contribution of exogenous Ra to the total Ra Phe to accurately calculate protein breakdown (but not protein synthesis). Exogenous Ra Phe includes only absorbed Phe that appears in peripheral blood where sampling occurs. Thus, absorbed Phe that is cleared on the first pass through the splanchnic bed will not contribute to the total Ra Phe. Hydroxylation of Phe to Tyr in the liver is the predominant site of splanchnic clearance. The measured fractional conversion of Phe to Tyr, which occurs only in the liver, applies to the absorbed Phe just as it applies to circulating Phe. Thus, exogenous Ra Phe can be calculated by correcting the amount of Phe absorbed by the fractional extraction by the liver. Two assumptions are required for this calculation: (1) the fraction of ingested beef that is absorbed, and (2) in the fed state there is no net change in splanchnic protein mass. We have discussed the implications of both of these assumptions previously [10].

The following equations were used for the calculation of whole-body protein kinetics:

- Total rate of appearance of tracer into plasma (Ra) (umol/kg/unit time) = *F*/*E*.
- Fraction of Ra Tyr from Ra Phe (fractional Ra of Tyr from Phe) = *E*_Tyr M+4_/*E*_Phe M+5_.
- Rate of phenylalanine hydroxylation to tyrosine (Phe hydroxylation rate) = Fraction of Ra of Tyr from Ra Phe × Ra Tyr.
- Fraction of Ra Phe hydroxylated to Tyr = Phe hydroxylation rate/Ra Phe.
- Protein synthesis rate (PS) (g protein/kg/unit time) = [(Ra Phe − Phe hydroxylation rate)/0.04] (note that this calculation is independent of the rate of appearance of exogenous Phe).
- Rate of appearance of exogenous Phe (ExoPhe) (μmol/kg/unit time) = [(g phe × TID) − Phe hydroxylation/0.8]/0.04.
- Protein breakdown rate (PB) (g protein/kg/unit time) = [(Ra Phe − Exo Ra Phe)/0.04].
- Net protein balance (NB) (g protein/kg/unit time) = Protein synthesis rate − protein breakdown rate.
- [11] (FSR, %/h) = [(*E*_BP2_ − *E*_BP1_)/ (Epl × *t*)] × 60 × 100.

Kinetic values were expressed per hour. In the fed state, the total response as compared to the fasting value was determined by calculating the total area under the curve of Phe and Tyr enrichments over the 5 h after meal ingestion. The unit of time for protein and amino acid kinetics in the fed state was thus 5 h. Enrichment (*E*) is expressed as tracer-to-tracee ratio (TTR) or mole percent excess (MPE), calculated as TTR/(TTR + 1). TTR was used for calculations of rates of protein breakdown whereas MPE was used for calculation of rates of protein synthesis. *E* is enrichment of respective tracers. *F* is the tracer infusion rate into a venous site. *E*_Tyr M+4_ and *E*_Phe M+5_ are plasma enrichments of tyrosine tracer at M + 4 and of phenylalanine tracer at M + 5 relative to M + 0, respectively. The correction factor of 0.04 is for conversion of phe kinetics to protein based upon the assumption that the contribution of phenylalanine to protein is 4% [10]. TID represents the true ileal digestibility, previously determined to be 0.90 for ground beef protein [9] and splanchnic extraction in the fed state was calculated by using phe hydroxylation divided by a hepatic dilution factor, 0.8 [12]. E_BP_ is the enrichment of muscle protein bound Phe and E_PL_ is the mean enrichment of plasma phenylalanine for two time points.

#### Analytical methods

Plasma samples were processed as previously described [13, 14]. Briefly, enrichments of phenylalanine and tyrosine were measured on the *N*-methyl-*N*-(tert-butyldimethylsilyl)trifluoroacetamide, Tertbutyldimetheylchlorosilane (MTBSTFA + 1% TBDMS; Regis Technologies, Morton Grove, IL, USA) derivative with the use of gas chromatography mass spectrometry (GCMS). Ions of mass to charge ratios of 234, 235, and 239 for phenylalanine and of 466, 467, 468, and 470 for tyrosine were monitored with electron impact ionization and selected ion monitoring. Muscle protein-free and -bound Phe were isolated and derivatized with MTBSTFA + 1% TBDMS reagent, and Phe enrichments were determined by GCMS by selectively monitoring ions of mass to charge 234 and 239 [13, 14]. Coefficients of variation for analysis of enrichment ranged from 0.5 to 3.0%.

Plasma amino acid concentrations were determined by liquid chromatography-mass spectrometry (QTrap 5500 MS; AB Sciex, Foster City, CA, USA) using the internal standard method, as described previously [13, 14]. The analytes were derivatized with 9-fluorenylmethoxycarbonyl chloride (Fmoc-Cl; 23186, St. Louis, MA, USA). Ions of mass to charge ratio of 340/144 for threonine, 338/116 for valine, 370/47 for methionine, 352/130 for isoleucine and leucine, 425/203 for tryptophan, 386/164 for phenylalanine, 598/154 for histidine, and 589/145 for lysine were monitored with selected ion monitoring on quadrupole one and three, respectively. Quantification of each peak was determined using MultiQuant software (version 2.1: AB Sciex). Coefficients of variation for analysis of amino acid concentrations ranged from 2.2% (threonine) to 5.4% (leucine).

### Statistical analysis

Statistical comparisons were made of the responses over the 5 h after meal consumption as compared to the corresponding basal value for each parameter. A 2-way repeated-measures of ANOVA was used to determined changes in plasma EAA, BCAA, and leucine concentrations and phe Ra over time. A two-tailed student’s *t *test was utilized to compare differences in protein kinetics (NB, PS, and PB), and muscle FSR. For a significant main effect or interaction, a two-tailed student’s *t *test or Fisher’s least significant difference test was performed for specific comparisons. *P* < 0.05 was considered to be statistically significant.

## Results

### Plasma amino acid concentrations and Ra Phe

**Fig. 3:**
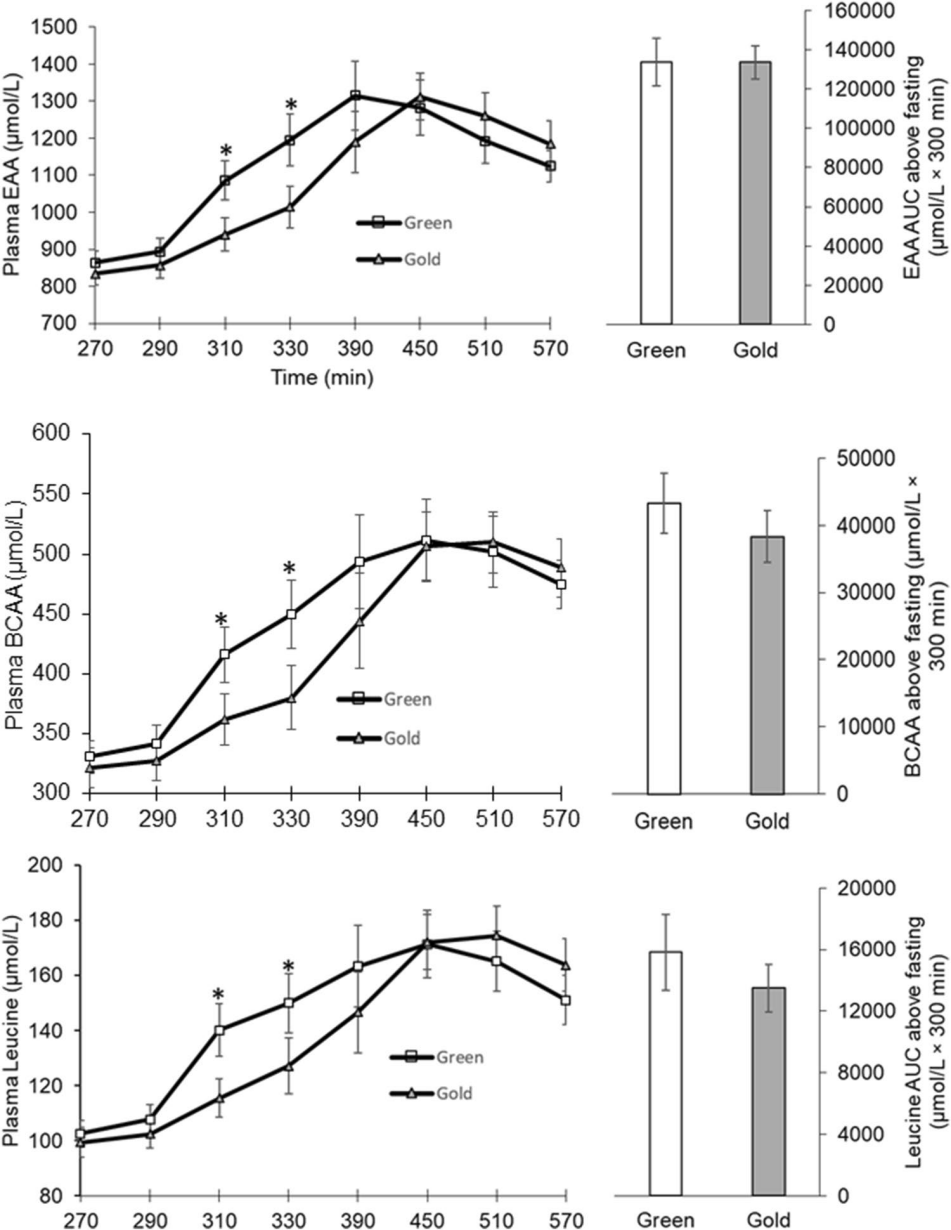
Plasma responses of total essential amino acids (EAA), branched chain amino acids (BCAA), and leucine following a meal of cooked beef and kiwifruit. There was a significant time by treatment interaction for EAA, BCAA, and leucine (*P* < 0.01). *Statistically significant between the kiwifruit treatment (*P* < 0.05). Values are expressed as means ± SE

**Fig. 4:**
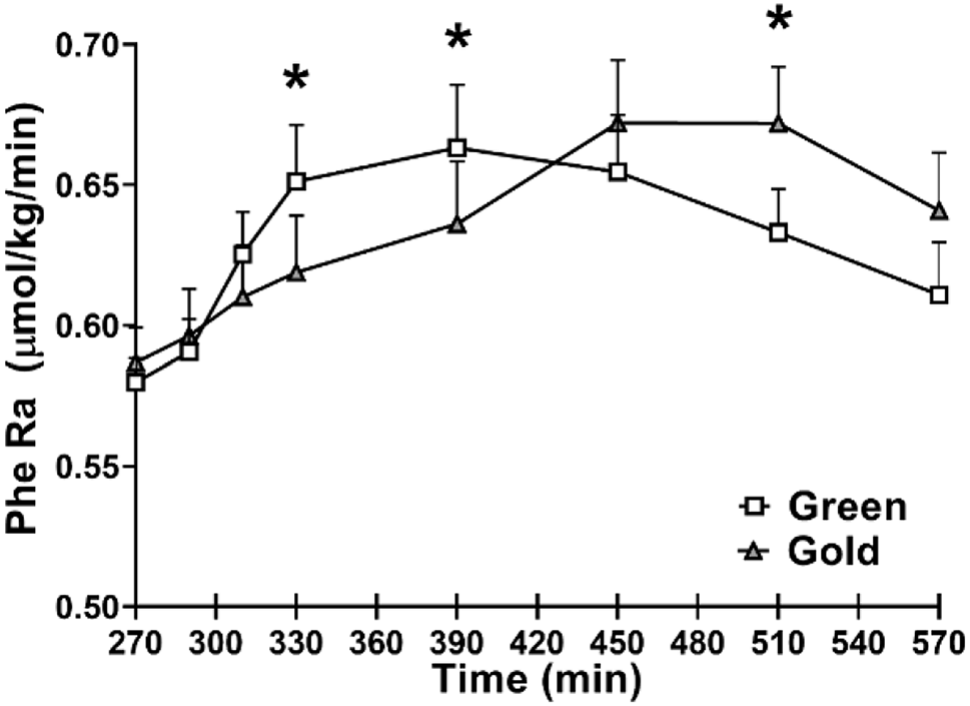
Total phenylalanine rate of appearance following the meal intake. *Statistically significant between green and gold kiwifruit treatment (*P* < 0.05). There was a significant interaction with time by kiwifruit variety (*P* < 0.01). Values are expressed as means ± SE

There were significant time by kiwifruit intake interactions for plasma concentrations of EAAs, branched chain amino acids (BCAAs), and leucine (*P* < 0.01) (Fig. 3). At 40- and 60-min following consumption of a test food, Hayward green kiwifruit induced greater plasma concentrations of EAA, BCAA, and leucine than Hort 16A gold kiwifruit (*P* < 0.01) (Fig. 3). Consistent with these observations, there was a significant time by intake interaction for Ra Phe (*P* < 0.01). Hayward green kiwifruit induced a greater Phe Ra at 60 and 120 min, while Hort 16A gold kiwifruit induced a greater Phe Ra at 240 min following consumption of the meal (Fig. 4). Although the rate of appearance of phenylalanine was greater at the earlier phase following Hayward green kiwifruit consumption in agreement with EAA concentration, the total area under the curve of these factors exhibited similar responses between groups (Fig. 4).

### Whole body and muscle protein kinetics

**Fig. 5:**
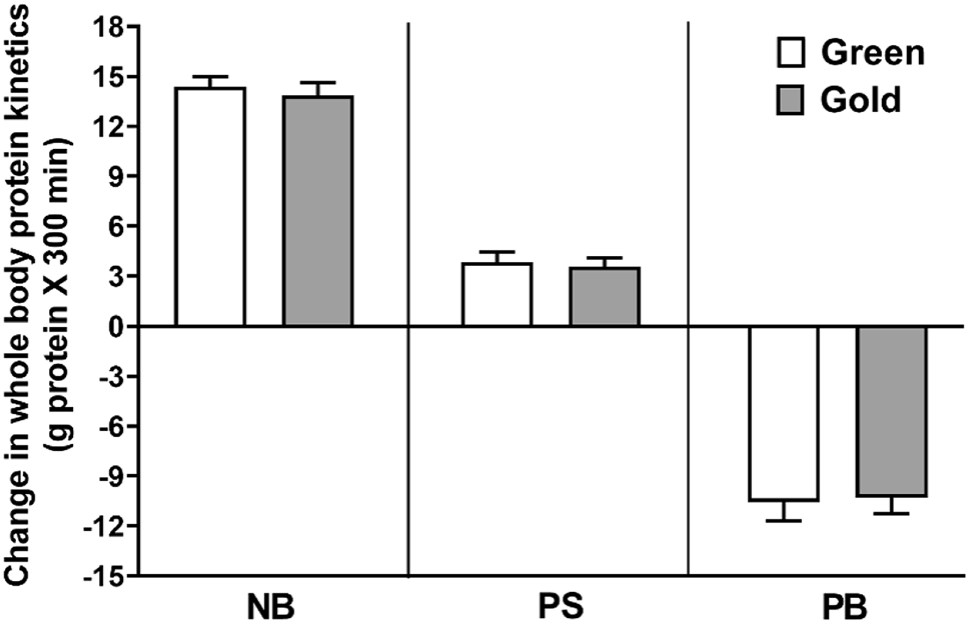
Whole-body protein responses [net balance (NB), protein synthesis (PS), and protein breakdown (PB)] over basal fasted values in response to meal intake containing 100 g beef and two green or gold kiwifruit. Values are expressed as means ± SE

There were no significant differences between Hayward green and Hort 16A gold kiwifruit with respect to integrated whole-body protein kinetics over the fed state (Fig. 5). Muscle fractional synthetic rate (FSR) increased to the same extent following consumption of each test food (data not shown). The numerical value were so similar that no reasonable number of subjects would had yielded significant differences in protein kinetics.

## Discussion

The primary effect of Hayward green kiwifruit, containing the actinidin protease, when co-ingested with cooked ground beef, was a more rapid transit of EAA to the peripheral circulation compared to Hort 16A gold kiwifruit. Hayward green and Hort 16A gold kiwifruit are very similar to each other in chemical composition, but the Hort 16A gold kiwifruit has minimal protease activity. The more rapid transit of EAAs following ingestion of the actinidin-containing Hayward green kiwifruit with beef was reflected by both a more rapid increase in plasma concentrations of EAAs, as well as a more rapid increase in exogenous Ra Phe. The more rapid increase in EAA concentrations and Ra Phe likely reflected a faster gastric emptying time. Although we did not measure this parameter in the current study, actinidin has been shown speed gastric emptying in the rat [6] and the pig [7].

It is likely that there were no differences in the responses of protein metabolism between the two types of kiwifruit is likely because the total amount of EAAs absorbed was similar. Results of previous studies support the concept that rapidly digested proteins (“fast” proteins) provide a greater anabolic stimulus (i.e., NB) than the same amount of “slow” proteins, or dietary proteins that are digested slowly [4]. However, in previous studies assessing the effect of speed of digestion on protein metabolism, not only did the plasma EAA levels increase at a greater rate after consumption of a “fast” dietary protein, but the peak EAA concentrations reached higher levels as well [15]. The importance of the extent of increase in plasma leucine concentration has been of particular interest (e.g. [16]). Since the true ileal digestibility of the amino acids in beef protein is high (90–97%) without any digestive aid [9, 17], minimal impact of Hayward green kiwifruit on total digestibility would be expected, even though the speed of digestion was enhanced. Alternatively, it may be that the difference in the speed of digestion between the two treatments was insufficient to affect protein kinetics.

The possibility exists that consumption of more than two Hayward kiwifruit would have had a greater impact on digestion of the beef meal. We chose to test the response to two kiwifruit with the meal because it is an achievable amount within normal consumption patterns. We used ground beef instead of beef steak to ensure homogeneous test meals in terms of nutrient profiles. Also, ground beef is commonly eaten by elderly to avoid chewing problems that may limit steak consumption [18]. However, ground beef has a greater digestibility than intact beef steak [5], and this may have minimized the effect of Hayward green kiwifruit on digestibility. It will be interesting in further work to study the effects of Hayward green kiwifruit consumption on whole meat and on poorer-cuts of meat with lower protein digestibility. Comparing the responses to Hayward green kiwifruit with other fruit that contain proteases, such as pineapple mango and papaya may provide insight. We used Hayward green kiwi fruit in the current study because evidence of effects on in vitro and in vivo animal digestion is more developed than for other fruits.

The technique we used to quantify whole body protein kinetics has limitations. The calculation of protein breakdown and NB required an assumption regarding dietary protein digestibility. We assumed that the true ileal digestibility of beef was the same in both treatments when considered over the entire post-prandial time frame, even though digestion was faster with the Hayward green kiwifruit. If true ileal digestibility was actually improved by the Hayward green kiwifruit, our calculations would have underestimated exogenous Ra Phe in that group. The underestimation of exogenous Ra Phe would result in an overestimation of PB, and thus an underestimation of the anabolic response, as reflected by NB. However, the true ileal digestibility of beef without co-ingestion of actinidin is 90% or more, so only a minimal improvement was possible. Consistent with this interpretation, neither the whole-body, nor muscle protein synthetic rates were improved by the Hayward green kiwifruit, and the total area under the curve for plasma EAA concentrations was similar with the two treatments as well.

In conclusion, consumption of Hayward green kiwifruit, containing the actinidin protease, enhances the speed of absorption of EAAs into the peripheral circulation following a beef meal in older individuals. Future studies could determine if enhanced absorption of EAAs decreases the feeling of fullness and ultimately the amount of beef consumption.

## Data Availability

The data are available upon written request.
