# Correction to: The impact of Hayward green kiwifruit on dietary protein digestion and protein metabolism

**DOI:** 10.1007/s00394-020-02432-9

**Published:** 2020-12-11

**Authors:** Sanghee Park, David D. Church, Carlene Starck, Scott E. Schutzler, Gohar Azhar, Il-Young Kim, Arny A. Ferrando, Paul J. Moughan, Robert R. Wolfe

**Affiliations:** 1 University of Arkansas for Medical Sciences, 4301 West Markham Street, Slot 806, Little Rock, AR 72205-7199 USA; 2 Riddet Institute, Massey University, Palmerston North, New Zealand; 3 Department of Molecular Medicine, Lee Gil Ya Cancer and Diabetes Institute, College of Medicine, Gachon University, Incheon, Republic of Korea

## Correction to: European Journal of Nutrition 10.1007/s00394-020-02363-5

The original version of this article unfortunately contained a mistake. Unfortunately, the Figs. 3 and 4 overlapped. Figures 3 and 4 should be:

**Fig. 3:**
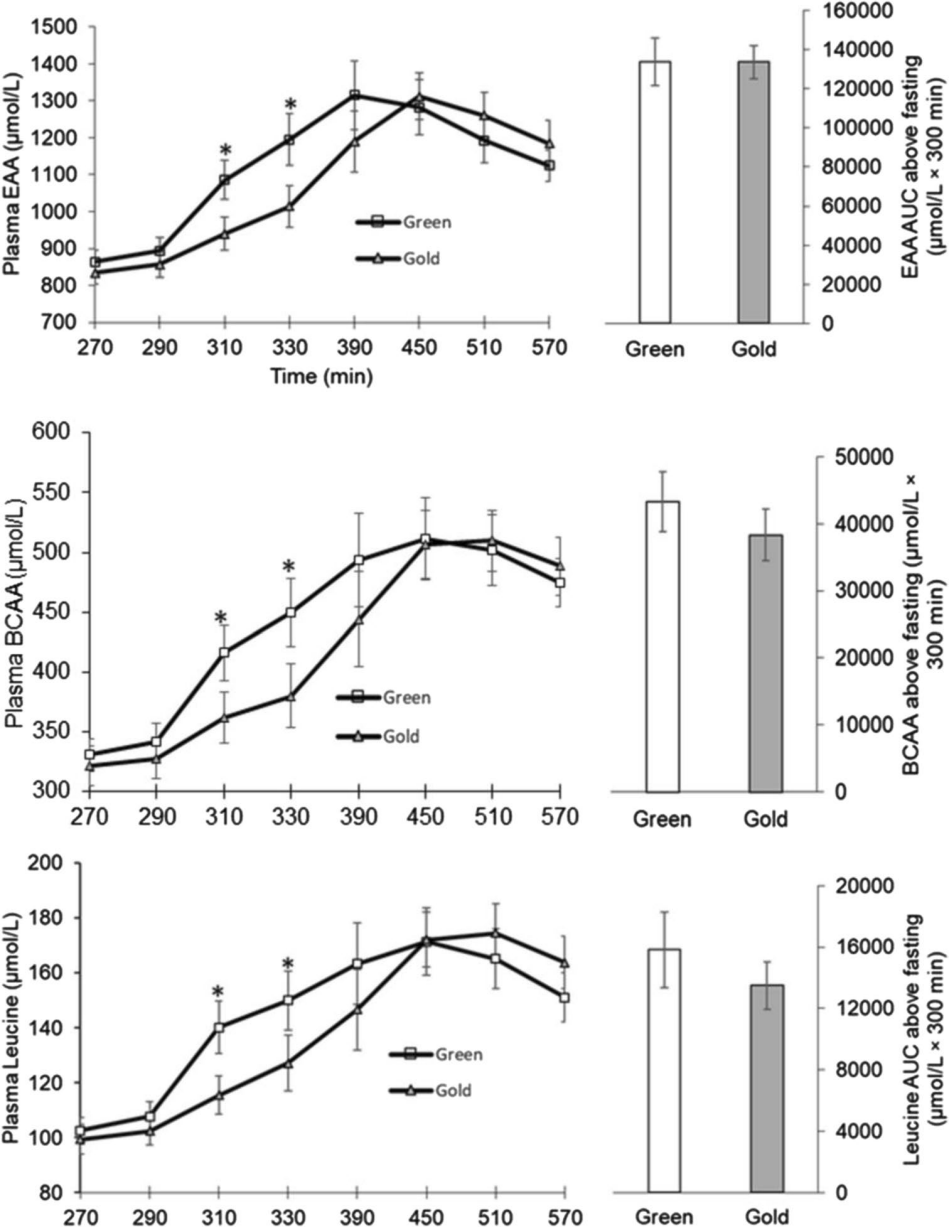
Plasma responses of total essential amino acids (EAA), branched chain amino acids (BCAA), and leucine following a meal of cooked beef and kiwifruit. There was a significant time by treatment interaction for EAA, BCAA, and leucine (*P* < 0.01). *Statistically significant between the kiwifruit treatment (*P* < 0.05). Values are expressed as means ± SE

**Fig. 4:**
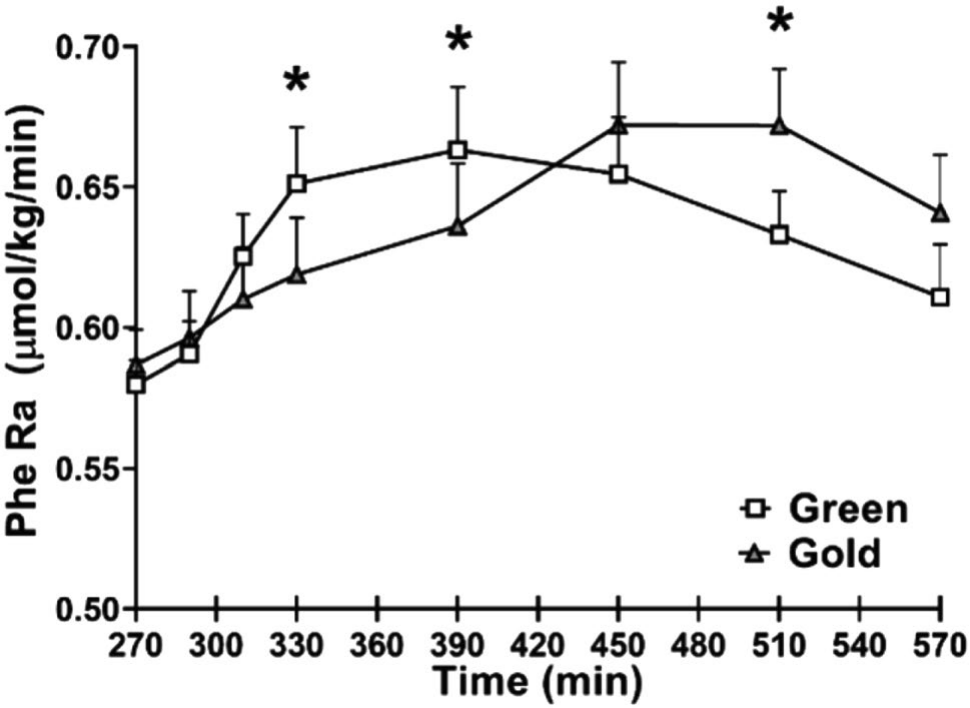
Total phenylalanine rate of appearance following the meal intake. *Statistically significant between green and gold kiwifruit treatment (*P* < 0.05). There was a significant interaction with time by kiwifruit variety (*P* < 0.01). Values are expressed as means ± SE

The original article has been corrected.

